# Araçatuba Virus: A Vaccinialike Virus Associated with Infection in Humans and Cattle

**DOI:** 10.3201/eid0902.020244

**Published:** 2003-02

**Authors:** Giliane de Souza Trindade, Flávio Guimarães da Fonseca, João Trindade Marques, Maurício Lacerda Nogueira, Luiz Claudio Nogueira Mendes, Alexandre Secorun Borges, Juliana Regina Peiró, Edviges Maristela Pituco, Cláudio Antônio Bonjardim, Paulo César Peregrino Ferreira, Erna Geessien Kroon

**Affiliations:** * Universidade Federal de Minas Gerais, Belo Horizonte, Brasil; † National Institutes of Health, Bethesda, Maryland, USA; ‡ Universidade Estadual Paulista–Araçatuba, São Paulo, Brasil; § Universidade Estadual Paulista–Botucatu, São Paulo, Brasil; ¶ Instituto Biológico, São Paulo, Brasil

**Keywords:** Araçatuba virus, cowpoxlike outbreak, emergent orthopoxvirus, TK, VGF, HA, and ATI genes, research

## Abstract

We describe a vaccinialike virus, Araçatuba virus, associated with a cowpoxlike outbreak in a dairy herd and a related case of human infection. Diagnosis was based on virus growth characteristics, electron microscopy, and molecular biology techniques. Molecular characterization of the virus was done by using polymerase chain reaction amplification, cloning, and DNA sequencing of conserved orthopoxvirus genes such as the vaccinia growth factor (VGF), thymidine kinase (TK), and hemagglutinin. We used VGF-homologous and TK gene nucleotide sequences to construct a phylogenetic tree for comparison with other poxviruses. Gene sequences showed 99% homology with vaccinia virus genes and were clustered together with the isolated virus in the phylogenetic tree. Araçatuba virus is very similar to Cantagalo virus, showing the same signature deletion in the gene. Araçatuba virus could be a novel vaccinialike virus or could represent the spread of Cantagalo virus.

The poxviruses comprise a family of large DNA viruses capable of infecting vertebrate and invertebrate hosts (*1*). Viruses from this family have caused naturally occurring or introduced infections in all populated continents (*2*). In Brazil, as in other parts of South America, little is known about the occurrence and circulation of poxvirus in the wild (*3*–*6*). After the worldwide elimination of smallpox in the 1970s, a few reports of poxvirus isolation in South America have been published, including scattered reports of parapoxvirus outbreaks in sheep and goat herds and virus isolation from wild or captive animals (*7*,*8*). The existence of mousepox outbreaks in animal facilities is also known, but most cases remain unpublished.

In recent years, however, many cases of unidentified diseases in dairy cattle with similar pathology have been reported in rural areas of Brazil, and some human infections have been associated with these illnesses in herds. Such diseases, characterized by the appearance of nodular and pustular lesions on bovine teats, are frequently related to viral infections such as bovine herpes mammillitis, pseudocowpox, and cowpox infections (*9*–*12*).

After clinical and initial laboratory analysis, cowpox virus (CPXV) was considered to be the obvious etiologic agent causing this human and cattle infection. CPXV (genus *Orthopoxvirus*) is the causative agent of localized and painful vesicular lesions. The virus is believed to persist in wild host reservoirs (including mammals, birds, and rodents), cattle, zoo animals, and domestic animals, including cats in parts of Europe and Asia. Contact of these reservoirs with susceptible animals and people can trigger the onset of disease (*13*,*14*). When humans are affected, the lesions occur on the hands and sometimes on the arms, usually followed by axillary adenopathy (*15*). However, CPXV isolation has not been reported from cattle or humans in Brazil, which led investigators to consider the possibility that infections were caused by vaccinia virus (VACV), since VACV was used as a live smallpox vaccine throughout the country until the late 1970s.

The occurrence of VACV-infected animals (domestic or wild species) is believed to be a result of contact with people recently vaccinated against smallpox. In fact, during mass smallpox vaccination campaigns, VACV infections were occasionally transmitted from the vesicular lesion on the vaccinee to domestic animals, usually cattle. In turn, infected animals transmitted VACV to susceptible people (*14*,*16*,*17*). Such infections were shown to be reproducible in experimental conditions (*18*).

Vaccinialike viruses have been isolated from the wild in Brazil; at least one of these viruses, the Cantagalo virus, was specifically obtained from infected cattle and humans after an outbreak of a cowpoxlike disease (*6*,*14*,*19*). These facts indicate the long-term establishment and active circulation of different vaccinialike viruses in the wild in South America, similar to the well-documented establishment of buffalopox virus in India (*19*,*20*).

We describe the isolation and characterization of a vaccinialike strain linked to a cowpoxlike outbreak affecting a dairy herd and associated with human infection; a similar outbreak attributed to Cantagalo virus infection was recently described (*14*). The virus reported here, named Araçatuba virus, was readily identified as a poxvirus by conventional methods, including characterization of pock morphology on the chorioallantoic membrane of chick embryos and electronic microscopy, which allows a quick differentiation between CPXV, pseudocowpox virus, and herpesvirus. However, such techniques do not differentiate between closely related viruses such as CPXV and VACV. To obtain accurate phylogenetic information, we detected poxvirus-conserved genes, such as thymidine kinase (TK), vaccinia growth factor (VGF), and hemagglutinin (HA), in the genome of Araçatuba virus using polymerase chain reaction (PCR). These genes were sequenced and the data used to generate phylogenetic trees. We also analyzed the A-type gene (ATI) based on restriction length polymorphism, which is a phylogenetic tool used to differentiate and classify orthopoxviruses (*13*). Based on these techniques, Araçatuba virus was shown to be similar to VACV–Western Reserve (WR) strain, the prototype member of the poxvirus family and the *Orthopoxvirus* genus. In addition, in relation to the HA gene, Araçatuba virus was very similar to Cantagalo virus, showing the same signature deletion in the gene. Such findings specifically point to the ubiquity of VACV circulating in the wild in Brazil as well as to the public health problems that may arise from the presence of this virus.

## Methods

### Case Report

**Figure 1:**
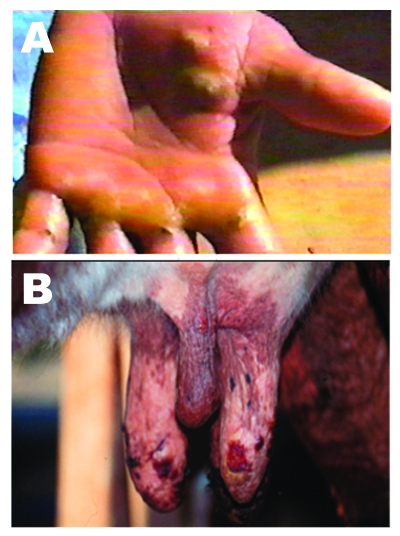
Lesions from suspected Araçatuba virus on hand of dairy farm worker (milker) (A) and teats of cow (B).

Five adult Girolanda cows from a herd of 40 animals were sent to the Veterinary Teaching Hospital at Unesp-Araçatuba, São Paulo State, Brazil; they had painful lesions on their teats, which interfered with milking. Lesions initially appeared on 2 cows and spread quickly to 35 animals, as well as the milker’s hands (Figure 1). Starting as a red focal area, the lesions developed quickly into a wound that healed with difficulty. No such episode had previously occurred on that farm. The cows had these symptoms for approximately 8 days before being taken to the veterinary surgeon. During the clinical examination, lesions in different stages were recognized; in most of the cows, nodular ulcerative wounds of 2–6 mm in diameter were predominant. Lesions were localized only on teats and udder, and many of them had dark, raw crusts. The teats had increased local temperature and were sensitive to touch. Because of the pain, cows avoided their suckling calves. At the farm, the only manual milker was also affected. The milker had >10 lesions on both hands and arms, but he did not initially accept any medical help and did not consent to examination. Because asepsic measures were not carried out, contact between the cows’ teats and the milker’s hands during milking probably enhanced the rapid spread of virus within the herd. Oral vesicles were not observed on calves’ muzzles or on buccal mucosae. Sterile samples of the vesicles and crusts were collected and sent to the Laboratório de Viroses de Bovídeos, Instituto Biológico, São Paulo for analysis. The animals were isolated from the herd, and teat lesions were treated with glycerine and a topical antibiotic, while the milker received medication at a nearby hospital. Three months after onset of infection, the remaining lesions on the cows were in an advanced healing process; however, all affected cows produced substantially less milk.

### Virus Isolation and Electron Microscopy

The material collected was prepared in 20% suspension of Eagle minimal essential medium (MEM) with 1% antibiotic to isolate the virus by inoculations in bovine fetal kidney cell monolayers at the Instituto Biológico, São Paulo. Samples that showed cytopathic effects were analyzed by transmission electronic microscopy. Material isolated from bovine fetal kidney cell monolayers was spread on the chorioallantoic membrane of embryonated chicken eggs and incubated at 37°C for 72 h (*21*).

### Cells and Viruses

VACV, WR strain, was obtained from the National Institute for Medical Research (Mill Hill, London, U.K.) and CPXV, Brighton strain, was provided by Dr. C. Jungwirth, Würzburg, Germany. Viruses were propagated in Vero cells and purified in a sucrose gradient as described (*22*). Vero cells were propagated at 37°C in MEM, supplemented with 5% fetal calf serum. Vero cells were also used for viral titration (*23*).

### Amplification and Cloning of Homologous VGF Gene and TK

The primers based on the TK and VGF nucleotide sequence of VACV–WR were produced as described by Fonseca et al. (*6*). The purified Araçatuba virus genome was used as a template, and temperatures of 45°C were used for annealing. Amplified fragments were cloned into the pGEMT vector (pGEM-T Easy Vector Systems, Promega Corp., Madison, WI). The portion of the HA coding sequence was amplified by using primers EACP1 and EACP2 as described by Roop et al. (*24*), and the approximately 900-bp fragment was produced and cloned into the pGEMT vector.

### Amplification and Restriction Fragment Length Polymorphism (RFLP) of ATI Gene

A PCR-based method for rapid screening and taxonomic differentiation is currently used to explicate *Orthopoxvirus* taxonomy (*25*,*26*). The assay uses primers designed from the ATI gene sequence from CPXV. We performed PCR with the primer pair ATI-up-1 5´AATACAAGGAGGATCT3´ and ATI-low-1 5´CTTAACTTTTTCTTTCTC3´. After the amplification reactions were carried out, the amplicons were digested with *Xba*I at 37°C for 3 h, as described (*26*).

### Nucleotide Sequencing

The PCR-amplified TK, VGF, and HA fragments of Araçatuba virus, cloned into the pGEMT plasmids, were sequenced in both orientations by the dideoxy-chain termination method (*27*) by using M13 universal primers (fmol DNA Sequencing System; Promega Corp.) and [α^32^ P]dCTP for oligonucleotide labeling. Sequences were analyzed by using the BLASTN and BLASTX programs (*28*). The DNA sequences of the Araçatuba virus, TK, and VGF genes were deposited in GenBank (accession nos. AF 503169 and AF503170). A phylogenetic tree was constructed by using the Treecon program with the Araçatuba virus–TK and Araçatuba virus–VGF nucleotide sequences (*29*).

## Results

### Virus Morphology

After Araçatuba virus was isolated in bovine fetal kidney cell monolayers, the samples were viewed by transmission electronic microscopy. Typical brick-shaped poxvirus forms were observed, measuring about 260 x 360 nm, with a superficial structure formed by tubules on long irregularly arranged filaments (data not shown). Samples were also added to embryonated chicken eggs so pock formations could be visualized on chorioallantoic membranes. White, nonhemorrhagic pocks were found (data not shown).

### PCR of Conserved Genes in *Orthopoxvirus* Genus and Nucleotide Sequence Analysis

**Figure 2:**
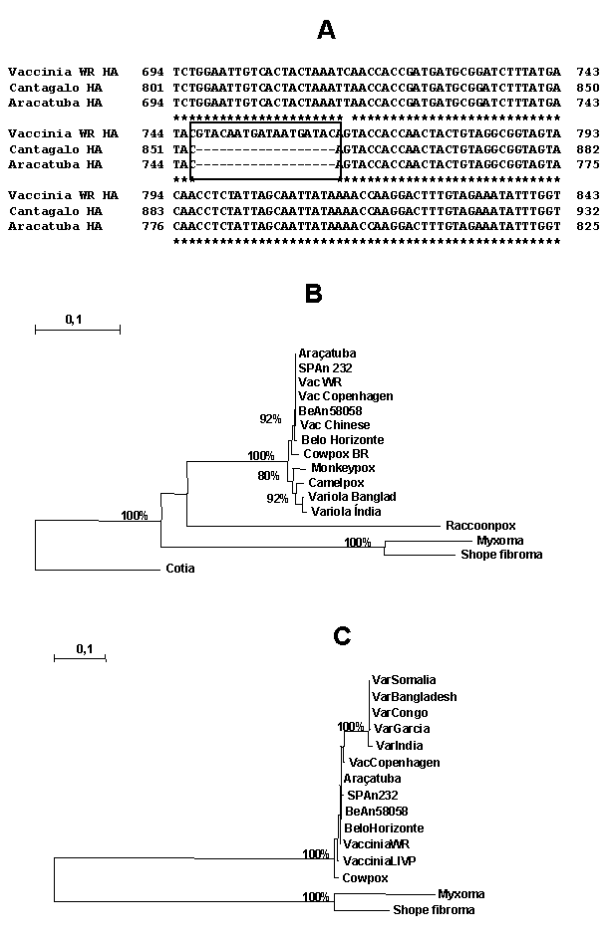
(A) Nucleotide sequence of the Araçatuba virus hemagglutinin (HA) and comparison with same sequences from Cantagalo virus and vaccinia virus–Western Reserve (WR). Box indicates deletion region conserved in the sequences of both Araçatuba and Cantagalo viruses, but not in vaccinia virus, Western Reserve (WR). Star (*) indicates regions conserved in all three viruses. (B) Phylogenetic tree constructed based on the nucleotide sequence of poxvirus thymidine kinase genes. Nucleotide sequences were obtained from GenBank (accession nos. X01978, M35027, M57768, AF163843, AF163844, EVY18384, U94848, K02025, S51129, L22579, S55844, X52655, and M14493). (C) Phylogenetic tree constructed based on the nucleotide sequence of poxvirus vaccinia growth factor genes. Nucleotide sequences were obtained from GenBank (accession nos. U18340, L22579, U18337, U18338, X69198, M35027, J02421, S61049, CVU76380, AF170722, and M15921). The Treecon program (29) was used to construct trees. Bootstrap confidence intervals are shown on branches (100 sample iterations).

PCR amplification of TK, VGF, and HA genes generated 528-, 381-, and 960-bp fragments, respectively. Amplicons were cloned into pGEMT vector and sequenced in both orientations. When compared to nucleotide sequences available in the GeneBank databases using the BLASTN program, the TK and VGF genes from Araçatuba virus were highly similar to homologous genes of VACV–WR. Optimal alignment showed similarity rates of up to 99.5% between Araçatuba virus and VACV–WR genes and minimal differences from nucleic acid substitutions. The coding region of HA gene was analyzed by alignment with similar sequences of VACV–WR and Cantagalo virus deposited in GenBank (accession nos. AF229247 and AF482758.1). The Araçatuba virus HA nucleotide sequence contained a signature deletion identical to a deletion detected in the sequence of Cantagalo virus (Figure 2A). This feature, absent in the sequence of most VACV strains, was used to correlate Araçatuba virus with VACV strain Istituto Ozwaldo Cruz (IOC), which was used as vaccine in some regions of Brazil during the smallpox eradication campaign (*14*). Using the nucleotide sequences from Araçatuba virus and other poxviruses, we constructed evolutionary trees with the Treecon program and placed Araçatuba virus isolate in the same cluster as other VACV strains (Figure 2B and 2C).

### Analysis of the ATI Gene Amplicom

**Figure 3:**
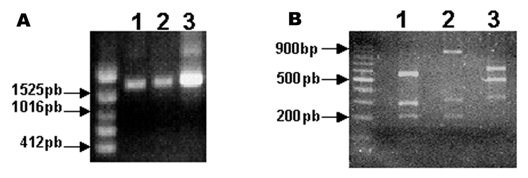
Detection and restriction fragment length polymorphism taxonomic analysis of the Araçatuba virus ATI gene. Primers based on the ATI gene nucleotide sequence from the cowpox virus were used to amplify the gene. (A) The amplified fragments were resolved on 0.6% agarose gel with ethidium bromide. Line 1 shows Araçatuba virus; line 2 shows vaccinia virus; and line 3 shows cowpox virus, Brighton strain. (B) Products obtained after amplification were digested with *Xba*I restriction enzyme. Fragments were resolved on 1.5% agarose gel stained with ethidium bromide. Arrowheads indicate molecular sizes (line 1, Araçatuba virus; line 2, vaccinia virus; line 3, cowpox virus, Brighton strain.

Although the formation of typical A-type inclusions is restricted to cells infected with cowpox virus, ectromelia virus, and raccoonpox virus (*2*), the sequence coding the N-terminus of the protein is highly conserved in many viruses, including CPXV, VACV, variola virus, camelpox virus, and ectromelia virus. These conserved sequences flank variable regions containing different size deletions, which may generate different size fragments after PCR amplification. The specificity of this assay is enhanced by the use of restriction enzymes, *Xba*I or *Bgl*II, allowing the detection of mutations at the restriction sites for these enzymes. We amplified the ATI gene from Araçatuba virus, VACV–WR, and CPXV for comparison. As described, the VACV–WR ATI amplicon generated 3 fragments after digestion with *Xba*I (*26*) (Figure 3). The larger fragment has approximately 900 bp, and the shorter fragments migrate closely, between the 300-bp and 400-bp markers. The profile obtained after digestion of Araçatuba virus ATI amplicon was similar to that of VACV–WR (Figure 3). The main difference, however, is that the larger fragment generated after *Xba*I digestion of the Araçatuba virus ATI amplicon is smaller than the VACV–WR fragments. These differences in size are also detected when nondigested ATI amplicons from Araçatuba virus and VACV are compared. Nevertheless, the pattern obtained for Araçatuba virus is completely different from the CPXV ATI pattern (Figure 3).

## Discussion

In Brazil, few studies have been conducted on the existence and circulation of poxviruses in the wild. In recent years, however, a growing number of poxvirus isolates have been obtained from samples from wild and domestic animals as well as humans; some of these viruses have caused cowpoxlike diseases in both animals and humans (*6*,*14*,*19*). All of these reports have shown that such viruses were related to VACV, which raises the question of whether populations of VACV are actively and widely circulating in the country among wild or domestic animal hosts. If so, such an event is similar to the history of the buffalopox virus in India and Southeast Asia. Until recently, that virus was considered an exclusive case of VACV being able to adapt to long-term survival in nature (*20*).

In this context, we isolated a novel virus, Araçatuba virus, from one of these cases of cowpoxlike diseases. The infection affected a herd of milking cows as well as their milker, in a rural area of the state of São Paulo, Brazil. Overall, our results suggest that the isolated virus is a VACV variant. Sequencing of conserved and nonconserved genes from poxviruses, such as TK, VGF, and HA, respectively, has been used for the classification of unknown poxvirus isolates (*6*,*14*,*19*). In the case of Araçatuba virus, phylogenetic trees designed from the nucleotide sequences of these genes indicate clearly that the virus belongs to the VACV subgroup like other orthopoxviruses isolated in Brazil during the 1960s and 1970s, the BeAn 58058 and Cotia viruses (*6*,*19*,*30*). This proposition is strengthened by RFLP analysis of the Araçatuba virus ATI homologous gene. This strategy has also been widely used for poxvirus taxonomy studies (*25*,*26*). Although the Araçatuba virus ATI pattern is not identical to the VACV–WR pattern, the virus fits on the VACV subgroup, and the pattern differs decidedly from the CPXV ATI pattern. Such differentiation is important because CPXV was the most obvious candidate to be the agent of such diseases. The Cantagalo virus ATI gene was characterized only at protein level and showed the same pattern of bands as the VACV strains (*14*).

For now, the discussion about the probable origin of Araçatuba virus, as well as other VACV isolated from animals and people in the country, is purely speculative. Araçatuba virus could be another vaccinialike strain or could represent the spread of Cantagalo virus. A logical assumption is to associate these viruses with variola vaccine stocks that may have escaped to the wild when the vaccination program was taking place in the 1970s and early 1980s. However, identifying the origin of those isolated VACV is difficult since many different samples, such as VACV-Lister, VACV-WR (Brazilian Health Ministry, pers. comm.), VACV-IOC (*14*), and even mixtures of different samples were used during the smallpox elimination campaign in Brazil. Researchers have proposed that at least one of the isolates, the Cantagalo virus, may have been derived from VACV-IOC (*14*). However, this finding is based on the nucleotide sequence of a single gene, and this issue is still a subject of some debate. Nevertheless, the Araçatuba virus HA nucleotide sequence revealed an interesting similarity with that of the same gene from Cantagalo virus, particularly at a signature sequence used to trace back the possible origin of this virus. Also of note, the Cantagalo virus was isolated in the city of Cantagalo (Rio de Janeiro state), about 850 km east of Araçatuba city. Moreover, a similar genetic feature of the HA gene was also detected in yet another cowpoxlike virus isolated from persons in the city of Muriaé (state of Minas Gerais), 800 km north of Araçatuba (data not published).

From the northern border at the Amazon region to the countryside of southeastern Brazil, an alarming number of genetically related vaccinialike viruses have been isolated from infected animals and humans. This fact clearly points to the existence and wide circulation of established, active VACV isolates in the vast wild and rural areas of Brazil. Whether the number of VACV infections has recently increased or whether only now they are being reported is difficult to determine. Nevertheless, the isolation of Araçatuba virus, together with other recently isolated viruses, was sufficient to trigger an alert by the Public Health Bureau in at least one of São Paulo’s neighboring states (Minas Gerais). How these viruses managed to persist in nature so long after the end of smallpox vaccination is a matter of speculation, but we think that they established circulation in some unknown wild hosts and were eventually transmitted to cattle and humans when they came in contact with populations of wild animals because of agricultural expansion.
